# About the complexity of adult onset Still’s disease… and advances still required for its management

**DOI:** 10.1186/s12916-016-0769-1

**Published:** 2017-01-06

**Authors:** Philippe Guilpain, Alain Le Quellec

**Affiliations:** 1 Montpellier 1 University, Medical School, Montpellier, F-34967 France; 2 Department of Internal Medicine – Multiorganic Diseases, University of Montpellier, Local Referral Center for Auto-Immune Diseases, Saint-Eloi Hospital, Montpellier, F-34295 France; 3 Inserm, U 1183, IRMB, Saint-Eloi Hospital, Montpellier, F-34295 France

**Keywords:** Adult onset Still’s disease, Macrophage activation syndrome, Score, Diagnosis, Prognosis, Therapy, Cytokine, Inflammasome

## Abstract

Adult onset Still’s disease (AOSD) is a rare inflammatory disorder that remains poorly understood. Its pathophysiology is yet to be completely elucidated, but is known to consist mainly on a cytokine cascade, responsible for the systemic manifestations. AOSD diagnosis is usually difficult and delayed, with physicians having to rule out several other conditions, including cancer or infectious diseases. Prognosis is heterogeneous and difficult to establish, ranging from benign outcome to chronic destructive polyarthritis and/or life-threatening events. In addition, treatment remains to be codified, especially considering the development of new drugs. In this commentary, we attempt to elucidate the complexity of AOSD and to highlight the need of working on prognostic tools for this disorder. We also discuss the numerous advances that would be useful for patients in the daily management of this disease.

Please see related article: http://bmcmedicine.biomedcentral.com/articles/10.1186/s12916-016-0738-8.

## Background

More than 100 years after its first clinical description, adult onset Still’s disease (AOSD) remains challenging to diagnose and treat, and an understanding of its pathophysiology is far from being achieved [1]. Even if the road ahead remains long and difficult, the study conducted by Ruscitti et al. [2] on the prognostic tools of AOSD represents a new step towards the better management of the disease. Their study, one of the largest published to date, provides confirmation of the impact of Pouchot’s ‘systemic score’ on AOSD prognosis. Indeed, these results are of significant importance considering that patient management is limited by disease complexity at the diagnosis, prognosis and therapeutic levels.

AOSD is a rare inflammatory disorder, the diagnosis of which is usually complex and delayed. Indeed, clinical presentation is heterogeneous and the main clinical features (spiking fever, joint involvement, skin rash and blood neutrophilia), as well as other minor ones (pharyngitis, lymph node or spleen enlargement, seritis, myalgia, hepatitis and abdominal pain), are unspecific [1]. In addition, histological confirmation and relevant biomarkers are lacking in this entity (notably, determination of glycosylated ferritin level is deceiving). Therefore, diagnosis is based on criteria (Yamaguchi’s or Fautrel’s) developed for classification rather than daily practice [3, 4]. In fact, AOSD has a diagnosis of exclusion and physicians have to rule out several other striking conditions (autoimmune, infectious or malignant diseases) through a complex diagnosis procedure. Conversely, in some cases, malignancies or infections have also been reported as triggering factors for AOSD, making the understanding of clinical presentation even more complex [1]. Disease complexity also lies in the pathophysiological mechanisms leading to a cytokine cascade (typically Th1) through an abnormal response of innate immune cells (especially macrophages) following the activation of Toll-like receptors [1].

Another complexity level is the highly variable prognosis, which ranges from a limited and benign outcome to chronic destructive polyarthritis and/or life-threatening involvement such as visceral complications (heart, kidney, lung or central nervous system), thrombotic microangiopathy or macrophage activation syndrome (MAS; also called reactive hemophagocytic lymphohistiocytosis) [1, 5–8]. Although exceptional, fatal events have been linked to AOSD-related complications and infections. Additionally, current treatments (i.e. NSAIDs, glucocorticoids and immunosuppressive drugs and biotherapies, in some cases) may also be deleterious, particularly in the long-term. Of note, the classification [5, 9, 10] distinguishing monocyclic, polycyclic and chronic AOSD is not useful for patient management, since it does not integrate severity and a functional and/or vital related prognosis. More precisely, it remains difficult to predict outcome at diagnosis and, consequently, drawing management guidelines presents a major challenge.

Clearly, joint involvement at diagnosis may affect functional prognosis through destructions resembling those of rheumatoid arthritis [11], while systemic manifestations can impair vital prognosis [6] (‘dichotomous view’ of AOSD [1]). A fever higher than 39.5 °C mainly occurs in systemic AOSD, whereas a leukocyte count higher than 30,000/mm^3^ and systemic inflammation markers are correlated with relapses [12]. Increased serum ferritin levels are associated with disease activity and chronic or recurrent forms of AOSD [7, 12–14]. In 1991, from a study on 62 patients, Pouchot et al. [5] proposed a ‘systemic score’ reaching up to 12 points, and assigning one point to each of the following manifestations: fever, skin rash, pleuritis, pneumonia, pericarditis, liver involvement, spleen involvement, lymphadenopathy, leucocytosis > 15,000/mm^3^, sore throat, myalgia, and abdominal pain. This score is easy to calculate, but remains to be validated in further studies.

In their article recently published in *BMC Medicine*, Ruscitti et al. [2] report that Pouchot’s ‘systemic score’ [5] successfully predicts a poor outcome in AOSD. More precisely, a ‘systemic score’ higher than 6 (discriminating cut-off ≥ 7) and the presence of any complication (MAS, kidney failure or myocarditis) at diagnosis are associated with mortality. Ruscitti et al. [2] observed MAS in 13 out of 100 patients recruited in the last 16 years, which is consistent with values in previous studies (10–15%) [8, 15]. However, death occurred in 10 patients, which is extremely high and unexepected. In AOSD, MAS is classically associated with persistent and/or refractory diseases and has been known to impair vital prognosis, but not at this level. For example, a recent Korean study identified 21 MAS among 109 AOSD patients and only two patients died [15], which is consistent with other published studies [8, 16]. In another recent study in Japan, the MAS-related mortality rate was approximately 20% among AOSD patients [17]. Therefore, the high mortality rate reported by Ruscitti et al. [2] undoubtedly has an important weight on the results, especially for the prognostic score, but also simply reflects on the potential severity of AOSD. Finally, since MAS is in reality a life-threatening event, this should not modify our perception of these findings.

## Conclusions

We hope that physicians will now be interested in Pouchot’s score and that further studies will simply confirm it as a prognostic tool. The discriminating cut-off predictive of death should also be evaluated and even precised. From this point of view, the study by Ruscitti et al. [2] represents an interesting step in the understanding of AOSD clinical presentation. Interestingly, these findings are in line with the ‘dichotomous view’ of AOSD [1] and could be helpful for the therapeutic decision between glucocorticoids alone or combined with immunosuppressive drugs/biologics as first-line treatment.

The final issue raised by this study concerns the methodology, whose improvement is crucial for clinical research on AOSD in the near future. In this rare inflammatory disorder, international or national (as that by Ruscitti et al. [2]) collaborative studies would provide the critical mass for pertinent statistical analyses and validation through evidence-based medicine.

As often observed, prognostic and therapeutic advances will probably arise from a better understanding of clinical phenotypes and pathophysiological mechanisms, and from a ‘dismemberment’ of AOSD into several entities, analogous to what ensued following the discovery of biomarkers, such as anti-neutrophil cytoplasm antibodies, on the spectrum of ‘polyarteritis nodosa’ and other vasculitides. The concept of AOSD as a disorder clinically meeting Yamaguchi’s criteria probably covers several entities with distinct pathophysiological processes but certain common inflammatory actors. Will the advances on inflammasome and cytokines help during the following years? Since AOSD is at the frontier of autoimmune and autoinflammatory disorders, the answer is ‘yes, probably’. In the end, what do the two forms of AOSD, namely systemic monophasic AOSD with high inflammation state at onset and complete recovery after several months versus chronic AOSD with systemic onset and chronic destructive sero-negative polyarthritis, have in common? The answer remains: ‘only our perplexity and ignorance…’
